# Grandparenting, health, and well-being: a systematic literature review

**DOI:** 10.1007/s10433-021-00674-y

**Published:** 2022-01-04

**Authors:** Mirkka Danielsbacka, Lenka Křenková, Antti O. Tanskanen

**Affiliations:** 1 Department of Social Research, University of Turku, Turku, Finland; 2 Department of Demography and Geodemography, Faculty of Science, Charles University, Prague, Czech Republic

**Keywords:** Custodial grandparents, Grandchild care, Grandparental health, Intergenerational relationships, Three-generation households

## Abstract

Whether grandparenting is associated with improved health or well-being among older adults is a salient question in present-day aging societies. This systematic review compiles studies that consider the health or well-being outcomes of grandparenting, concerning (1) custodial grandparent families, where grandparents are raising grandchildren without parental presence; (2) three-generation households, where grandparents are living with adult children and grandchildren; and (3) non-coresiding grandparents, who are involved in the lives of their grandchildren. Review was based on literature searches conducted in September 2019 via Web of Science, PubMed, PsycINFO, and Ebsco. We screened 3868 abstracts across four databases, and by following the PRISMA guidelines, we identified 92 relevant articles (117 studies) that were published between 1978 and 2019. In 68% of cases, custodial grandparenting was associated with decreased health or well-being of grandparents. The few studies considering the health or well-being of grandparents living in three-generation households provided mixed findings (39% positive; 39% negative). Finally, in 69% of cases, involvement of non-coresiding grandparents was associated with improved grandparental outcomes; however, there was only limited support for the prediction that involved grandparenting being causally associated with grandparental health or well-being. Despite this, after different robustness checks (counting all nonsignificant results, taking into account the representativeness of the data and causal methodology), the main finding remains the same: the most negative results are found among custodial grandparents and three-generation households and most positive results among non-coresiding grandparents.

## Background and objectives

Due to increased life expectancy, the proportion of older adults, including grandparents, has increased on a global scale, and in fact, it has been estimated that currently approximately 13% (one billion) of world population are grandparents (Moore and Rosenthal 2016). Most grandparents play an active role in the lives of their grandchildren. In Europe, for instance, 58% of grandmothers and 49% of grandfathers provide regular grandchild care (Hank and Buber 2009), while approximately 2% of children are raised by their grandparents in the USA meaning that one million grandparents in the USA are the primary caregivers for their grandchildren (Dunifon et al. 2014). Thus, grandparents are often highly involved in their grandchildren’s lives and whether grandparenting provides benefits or disadvantages for grandparents in terms of their health or overall well-being is a salient question.

Many studies that attempt to detect whether grandparenting is associated with improved health or well-being predict that grandparents benefit from being involved in their grandchildren’s lives (e.g., Mahne and Huxhold 2015; Tsai et al. 2013). A counterhypothesis, however, takes the stance that caring for young children is challenging, particularly for older adults with limited reserves of strength (e.g., Baker and Silverstein 2008a, b; Hughes et al. 2007). According to this perspective, active grandparenting could overburden older adults and lead to grandparents’ decreased health and well-being. For example, Coall and Hertwig (2010, 2011) argue that the association between grandparenting and grandparents’ health may result in an inverted U-shaped curve. Based on the Coall–Hertwig hypothesis, moderate grandparental involvement is the most beneficial for grandparents, while negative effects may arise when no grandparental involvement occurs or when it reaches the highest level of involvement (e.g., when grandparents are the primary caregivers of their grandchildren).

Thus, whether grandparenting improves the health or well-being of grandparents may depend on living arrangements that are related to the degree of grandparental involvement, which is why grandparents are commonly separated into three groups: (1) custodial grandparents, (2) grandparents living with their children and grandchildren in three-generation households, and (3) non-coresiding grandparents (i.e., those involved in their grandchildren’s lives without living with them). In households where the grandparents are the primary caregivers of their grandchildren or they live with their descendants in a three-generation household, the high level of their involvement is assumed based on the living arrangements. Among non-coresiding grandparents, however, grandparental involvement is most often measured via childcare support but also the frequency of contact, emotional closeness, and other informal assistance.

Cultural variation in living arrangements, filial norms, and grandparental involvement are substantial (Shwalb and Hossain 2017). Living in three-generation families or being a custodial grandparent is much more common in many Asian countries than in Western ones. This variation has been partly explained by the influence of Confucianism, which promotes a tradition of filial responsibility (Burr and Mutchler 1999; Speare and Avery 1993). Also reciprocity may be an important factor in Asian countries with strong filial obligations (e.g., Sheng and Settles 2006). Cultural traditions could also influence on whether custodial grandparenting or living in three-generation household is associated with positive or negative outcomes among grandparents because in Asian countries grandparents living with grandchildren are not as selected group as they are in Western countries. In addition, due to the lack of publicly provided old age support grandparents need to rely on their children and thus living with them or with grandchildren could provide benefits to grandparents themselves. The expectation is, that the negative effects of highly involved grandparenting observed in Western countries are not present or could be even positive in Asian countries.

This review makes a novel contribution to the literature by compiling research on all three contexts of grandparenting and revealing how in each context the involved grandparenting is associated with grandparental health or well-being. Cultural context of grandparenting is taken into account as the review observes also the distribution of studies and results by various countries. In addition, the review investigates whether previous studies have provided convincing causal evidence for the possible association.

### Aim of the review: to reveal a grandparenting effect

Our main aim is to investigate whether grandparenting (or grandparental involvement) is associated with the health or well-being of grandparents and whether this association is positive or negative. Grandparent outcomes have been measured with several variables which we can summarize into two rough categories: health and well-being. The health category includes, for instance, longevity, cognitive skills, mental health, depressive symptoms, stress levels, physical health, frailty index, self-rated health, preventive health behavior, and limitations in activities of daily living. Well-being category in turn includes variables such as happiness, life satisfaction, subjective well-being (SWB), and perceived quality of life. Of course, these broad categories are not mutually exclusive but rather interrelated. Being in good health is probably associated with increased well-being and vice versa. Studies detecting the effect of grandparental involvement on grandparent outcomes have commonly used one or several of these outcomes and to be as comprehensive as possible, we try to take all such studies into account.

Three contexts of grandparenting (i.e., custodial grandparents, grandparents in three-generation households, and non-coresiding grandparents) have been considered, respectively. In addition, the geographic and cultural context of grandparenting has been observed. As we conducted a database of studies included in the review, we marked each study’s result as being either positive, negative, or nonsignificant, depending on the association and its statistical significance between grandparents’ involvement and their health or well-being. Some studies that included more than one context of grandparenting may have been marked as providing nonsignificant results in one context (e.g., custodial grandparents) but positive results in another (e.g., non-coresiding grandparents; Choi and Zhang 2018). Likewise, some studies may provide negative results in one context and positive results in another (e.g., Hughes et al. 2007).

In several cases, more than one health or well-being outcome was investigated in a single study. We marked the result of a study as being either positive *or* negative, even if there was one positive or negative association revealed, and we marked a study as being both positive *and* negative if it contained both results. The latter was often the case if the results were separated according to gender (e.g., Hughes et al. 2007) or ethnicity (e.g., Goodman and Silverstein 2002, 2006). Also, a study was marked as nonsignificant if *all* the results in specific grandparent groups showed nonsignificant associations (e.g., Ates 2017; Hsu and Chang 2015). Thus, the total number of positive, negative, or negligible results exceeded the number of studies included in the review (see Table 2). However, in the review, we also counted the total number of *all* the results in the studies, which was substantially higher than counting only the positive, negative, and nonsignificant results overall, as one study may have several positive, negative, or nonsignificant results due to multiple outcome measures and separations (see Table 4).

## Research design and methods

### Search strategy

On September 27, 2019, we conducted a systematic literature search in three databases: Web of Science, PubMed, and PsycINFO. Then, on September 30, 2019, we included one more database, Ebsco, in the review process. We limited the search to peer-reviewed articles in English that employed a quantitative method and were published between 1970 and 2019. In practice, the first study in our sample is from 1978 (Wood and Robertson 1978) because before this there were none eligible studies.

Our search words included the following familial circumstances or terms related to grandparenting: intergeneration*; multigeneration*; “custodial grandparent*”; three-generation*; “skipped generation*”; grandchild*; “extended family*”; “extended household*”; alloparent*; “co residence”; co-residence, coresidence, grandparent*; grandmother*; grandfather*; grandmaternal*; and grandpaternal*. We also included search words related to grandparental investment, health or well-being: care; “primary care*”; cognition*; “mental health”; depression, depressive; “physical health”; “self-rated health”; “self-rated health”; “activities of daily living”; ADL; happiness; and “life satisfaction.”

### Screening eligibility and inclusion criteria

**Fig. 1:**
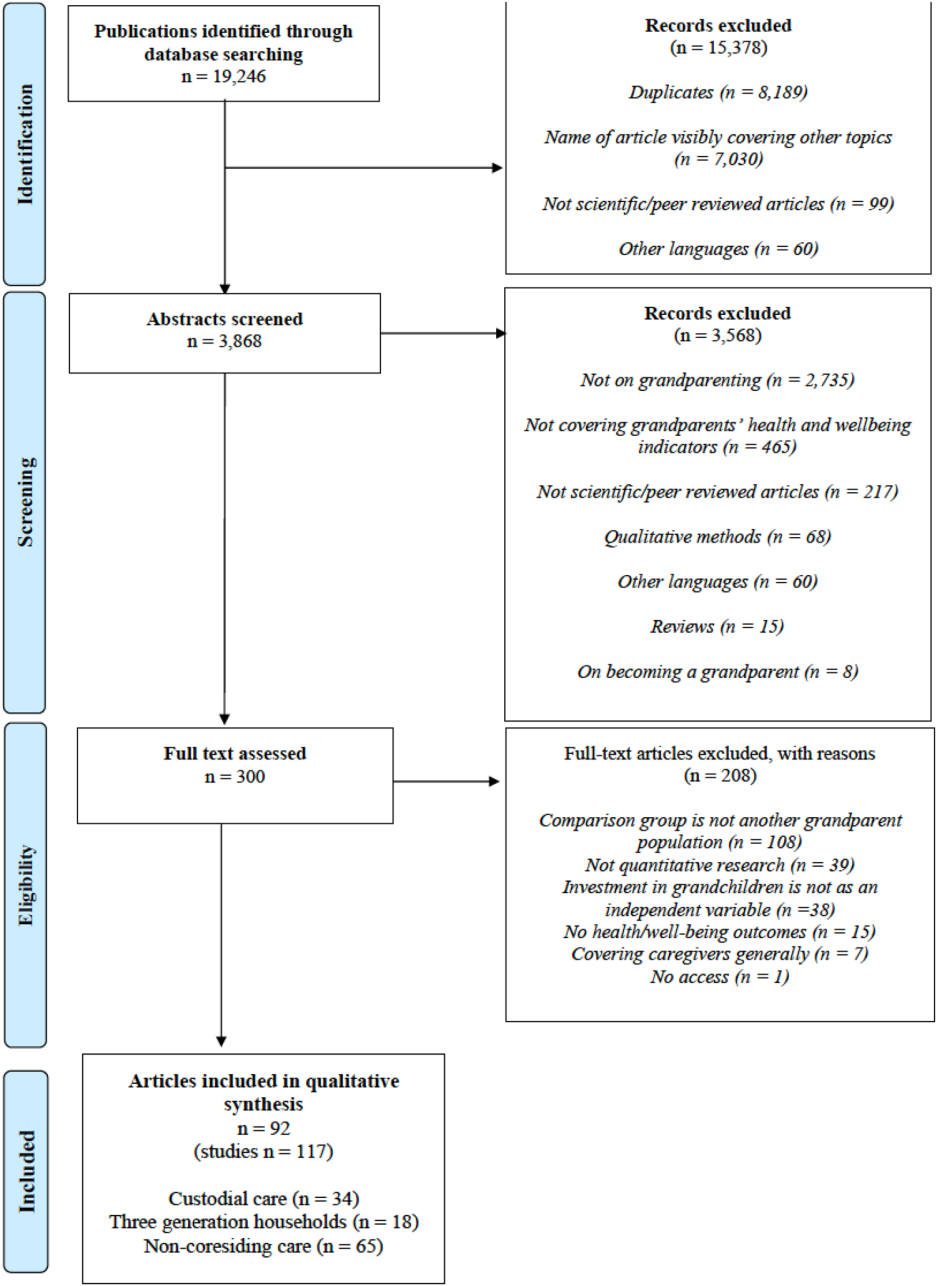
PRISMA 2009 flow diagram. From: Mother D, Liberati A, Tetziaff J, Altman DG, The PRISMA Group (2009). Preferred Reporting items for systematic reviews and Meta-analyses: The PRISMA statement. PLoS Med 6(7): e1000097. doi: 10.1371/journal.pmed1000097. For more information, visit www.prisma-statement.org.

The review’s search yielded 19,246 records in total, but we excluded the following articles: duplicates (*n* = 8189), those that covered other topics based on the article’s name (*n* = 7030), those that were not scientific or peer-reviewed (*n* = 99), and those that were in other languages besides English (*n* = 60). Thus, we included 3868 abstracts for screening, and afterward, we excluded articles that did not concern grandparenting (*n* = 2735) or include indicators about grandparental health or well-being (*n* = 465). We also excluded those that were not peer-reviewed (*n* = 217), only employed qualitative methods (*n* = 68), were in other languages besides English (*n* = 60), were reviews (*n* = 15), and those specifically concerned with being/becoming a grandparent (*n* = 8) (Fig. 1).

We assessed the full text for 300 articles, excluding those that were not based on quantitative research (*n* = 39), did not have grandparental investment as an independent variable (*n* = 38), included no grandparental health or well-being outcomes (*n* = 15), and those that only generally covered caregivers but did not distinguish between grandparents and other types of caregivers (*n* = 7). Finally, we excluded studies that only concerned custodial grandparents (*n* = 99) or only three-generational households (*n* = 9) if they did not include a comparison group of either non-custodial or non-coresiding grandparent populations. The exceptions included studies that compared grandparents’ health or well-being before and after they were custodial grandparents or before and after they began living in three-generational households. Finally, one study could not be accessed, and based on the abstract, we were unable to evaluate whether it should have been included in the review (Minkler and Fuller-Thomson 2001).

Based on these selections, a total of 92 *articles* (i.e., peer-reviewed, published research reports) were included in the final sample. We classified all the *studies* (i.e., part of an article that covers one of the abovementioned types of familial circumstances) based on whether they concerned custodial grandparents (*n* = 34), three-generational households (*n* = 18), or non-coresiding grandparents (*n* = 65). Some articles covered more than one type of family circumstance; thus, the total number of studies included was higher (*n* = 117) than the number of articles in the final database. In this paper, the term, *result*, refers to a single finding that concerns grandparental involvement and an outcome measure (whether article had at least one positive and/or negative or negligible result *n* = 141; all results *n* = 452). In this sense, an article can contain a maximum of three studies, while a single study can contain several results.

## Results

### Descriptive findings

The populations that were studied in the review are shown in Table 1. Most research was conducted with data from the USA (*n* = 48), especially in the custodial grandparent group. Also, many studies, especially in the non-coresiding grandparent group, were conducted with data from European countries (*n* = 21), and of these, a significant number utilized data from multiple European countries by using the Survey of Health, Ageing and Retirement in Europe (SHARE) data (*n* = 12). However, studies using data from European countries are missing from the custodial grandparent group, which is likely because the number of custodial grandparent households is much lower in Europe (particularly Western Europe) than in the USA or Asian countries (Shwalb and Hossain 2017). In addition, there were 29 studies utilizing data from Asian countries, 7 studies from Australia, 2 from South America, and 10 from other countries (4 from Turkey, 4 from Kenia and 2 from Israel).

**Table 1:** Number of studies by study population and family circumstances, total 117

Population	Grandparent type			Total
	Custodial	Three-generation	Non-coresiding	
USA	23	10	15	48
Finland	0	0	1	1
Sweden	0	0	1	1
Germany	0	0	4	4
Ireland	0	0	2	2
Spain	0	0	1	1
Europe	0	0	12	12
China	1	2	5	8
South Korea	2	1	6	9
Taiwan	1	4	5	10
Thailand	1	0	1	2
Australia	1	0	6	7
Chile	0	0	1	1
Mexico	0	0	1	1
Israel	0	0	2	2
Kenya	4	0	0	4
Turkey	1	1	2	4
Total	34	18	65	117

### Custodial grandparents

Custodial grandparents are the primary caregivers for their grandchildren, and recently, the number of these “skipped-generation households,” or “grandfamilies,” has increased in many Western countries. Currently, this population group is highest in the USA, where approximately 2% of children are raised by their grandparents (Dunifon et al. 2014). While grandparents may be responsible for raising their grandchildren for many reasons, among the most common in Western countries are parental teenage pregnancy, drug addiction, mental/physical health problems, incarceration, distance employment, relationship breakdown, and death (Hayslip et al. 2017). In Asian countries, however, grandparents mostly raise their grandchildren due to distance employment, especially in China, where parents often leave their children with their parents due to temporary migration, which refers to working in other locations (Chen and Liu 2012; Cong and Silverstein 2008).

In the review, a total of 68% (23/34) of the custodial grandparent studies were conducted with data from the USA (Table 1 and Appendix Table 6). According to the research describing custodial grandparents’ characteristics in the USA, they are more often concerned with women than men, and more often, they focus on the maternal side (Dunifon et al. 2014; Hayslip et al. 2017). In most cases, custodial grandparents in the USA are members of lower socioeconomic classes, single women (Fuller-Thomson et al. 1997; Heywood 1999; Minkler and Fuller-Thomson 2000), African-Americans, and between ages 50 and 59, whereas only very few are under 40 or over 80 (Ellis and Simmons 2014).

Thus, in the USA, custodial grandparent families are predominantly a selected group, which inevitably affects any comparison between custodial grandparents and non-custodial grandparents. According to the studies in this review, grandparents raising grandchildren have a higher risk of various health problems. Among 68% (30/44) of the results, the health or well-being of custodial grandparents was, at least in some grandparent subpopulations, poorer than non-custodial grandparents, their counterparts (Table 2 and Appendix Table 6). For instance, custodial grandparents have a higher risk of being limited in their daily activities as well as having depressive symptoms, elevated stress levels, and poorer self-rated health than their counterparts (e.g., Baker and Silverstein 2008a; Blustein et al. 2004; Minkler and Fuller-Thomson 1999, 2005; Musil et al. 2011). Most studies in this group have used measurements related to grandparental health but also some used measurements related to well-being (e.g., life satisfaction, quality of life) and also these revealed mostly negative associations (Bowles and Myers 1999; Wilmoth et al. 2018; Yalcin et al. 2018). Many of the detrimental effects on grandparents’ health or well-being in skipped-generation households are likely due to their characteristics and history rather than their caring responsibilities exclusively.

**Table 2:** Summary of associations in grandparent groups: custodial, three-generation households, and non-coresiding

	Positive association	Negative association	No association	Total no. of studies	Total no. of results
Custodial care	12 (27%)	30 (68%)	2 (5%)	34	44
Three-generation	9 (39%)	9 (39%)	5 (22%)	18	23
Non-coresiding	51 (69%)	14 (19%)	9 (14%)	65	74
Total	72 (51%)	53 (38%)	16 (11%)	117	141

Number of results and % of total number of results

Although many studies on custodial grandparents have been conducted with cross-sectional data and could reveal selection effects, numerous investigations also contain longitudinal data (*n* = 16 studies, *n* = 21 results; Table 3). In these studies, a negative association is also apparent, as a grandparent who begins raising a grandchild often suffers from a decline in health (e.g., Baker and Silverstein 2008a, b; Musil et al. 2011). Thus, it might not only be selection that explains the negative association between custodial grandparenting and grandparental health.

**Table 3:** Summary of associations in custodial, three-generation, and non-coresiding grandparent groups with longitudinal data and/or causal modeling

	Positive association	Negative association	No association	Total no. of studies	Total no. of results
Custodial care	7 (33%)	12 (57%)	2 (10%)	16	21
Three-generation	4 (31%)	6 (46%)	3 (23%)	11	13
Non-coresiding	26 (72%)	6 (17%)	4 (11%)	32	36
Total	37 (43%)	24 (34%)	9 (13%)	59	70

Number of results and % of total number of results

Among the results, only 27% (12/44) showed positive outcomes for custodial grandparents (Table 2 and Appendix Table 6). Twelve studies report at least one positive result between being a custodial grandparent and grandparental health, including those from the USA (7), South Korea (1), Taiwan (1), Kenya (2), and Thailand (1). Most of these studies, however, also report some negative results regarding an outcome or grandparent group (Appendix Table 6), and importantly, only two studies report solely positive results (Chung and Park 2018; Ku et al. 2013).

Within this category about half of the studies were conducted with representative data (47%, 16/34) and half with non-representative data (53%, 18/34). Among positive results 50% (6/12), the data used were representative and among negative results 41% (13/32).

In summary, there are two options for interpreting the results, which showed that, in most cases, custodial grandparents reported poorer health when compared to their non-custodial counterparts. The first option is that a decline in health occurs when one enters the role of a custodial grandparent since it increases the burden of caregiving. However, one study shows that grandmothers, who had been raising and continued to raise their grandchild, were more likely to have preventive health behaviors (Baker and Silverstein 2008b), meaning that the health decline may not be considered long-lasting. The second option is that these findings are based on selection effects, and custodial grandparents—especially in Western societies—are typically considered part of a disadvantaged group. In Asian countries, however, custodial grandparents do not constitute a disadvantaged group, so the results may differ. In this review, 5/34 studies concerning custodial grandparents were conducted with data from Asian countries, and the results were either nonsignificant (Chen and Liu 2012; Choi and Zhang 2018), positive (Chung and Park 2018; Ku et al. 2013), or both positive and negative (Komonpaisarn and Loichinger 2019).

### Grandparents in three-generation households

The terms, “three-generation” and “multigenerational” families, refer to a living arrangement whereby children, parents, and grandparents live together in the same household. The number of three-generation households varies remarkably between countries. For instance, approximately 25% (or more) of adolescents in Southern European countries live in three-generation households, whereas the number of children living in multigenerational households is less than 5% in Scandinavian countries (Kreidl and Hubatkova 2014). Meanwhile, by age five, almost a fourth of children in the USA live in three-generation families, while 8% and 11% do so in the UK and Australia, respectively (Pilkauskas and Martinson 2014).

Few investigations examine whether living in three-generation households is associated with improved or impaired outcomes among grandparents, (Dunifon et al. 2014) and, similar to custodial grandparenting, this population group is primarily studied in the USA (Dunifon et al. 2016). In our review, 56% (10/18) of the studies were conducted with US data, while 39% (7/18) was data from Asian countries and one study included data from Turkey.

Based on the results, living in a three-generation household is as likely to be beneficial as detrimental for grandparents. Of the results, 39% (9/23) showed a positive association, but 39% (9/23) also showed a negative association. Meanwhile, 22% (5/23) of the results showed a nonsignificant association (Table 2 and Appendix Table 7). Many of the articles examining grandparents’ health or well-being while living in three-generational households also consider custodial grandparents, so in these cases, comparisons are often made between these two groups (e.g., Blustein et al. 2004; Goodman and Silverstein 2002; 2006). The outcome measures that were utilized include, for instance, the following that can be counted as health measurements: depressive symptoms, self-rated health, functional/mobility limitations, and different stress factors and the following that can be counted as well-being measurements: happiness, quality of life, and life satisfaction (e.g., Tsai et al. 2013; Musil and Ahmad 2002; Ku et al. 2013; Hsu and Chang 2015; Goodman 2003; Yalcin et al. 2018).

Like the results concerning custodial grandparents, those involving grandparents in three-generation households may reflect the selection effect, meaning that grandparents living in three-generation households may already have poorer health than those in different living arrangements (Hughes et al. 2007). However, studies with longitudinal data (*n* = 11 studies, *n* = 13 results; Table 3) indicate that negative (e.g., Chen and Liu 2012; Hughes et al. 2007; Musil 2000) and positive (Tsai et al. 2013; Musil et al. 2011; Hughes et al. 2007) associations exist, even when the health or well-being of a grandparent is measured over time.

Positive and negative results were found in studies that were conducted with data from both the USA and Asia (e.g., Chen et al. 2015; Chen and Liu 2012; Hughes et al. 2007; Tsai et al. 2013). Thus, the positive/negative results were not solely related to the country of residence; however, the results that were solely positive were more often found in studies with data from Asian countries (Guo et al. 2008; Ku et al. 2013; Tsai et al. 2013). Among three-generation household studies, 44% of them were conducted with representative data (8/18) and 56% with non-representative data (10/18). Among positive results 33% (3/9), among negative results 33% (3/9), and among nonsignificant results 60% (3/5), the data used were representative.

### Grandparents living separately from their grandchildren

The largest group of caregiving grandparents, particularly in Western countries, includes those who do not live with their grandchildren but provide them with different kinds of support relatively frequently. In recent decades, an increasing number of studies have investigated the associations between active grandparenting and the health and well-being of non-coresiding grandparents. Most studies that focus on non-coresiding grandparents are conducted with European data (32%; 21/65), and over half of these (12 studies) contain data from multiple European countries. Meanwhile, 26% of the studies (17/65) were conducted with data from Asian countries, 23% (15/65) utilized data from the USA, 9% (6/65) utilized data from Australia, and the remaining 9% (6/65) involved data from other countries (e.g., Israel or Turkey) (Table 1 and Appendix Table 8).

Non-coresiding grandparents are involved in the life of their grandchildren in many ways, and the most common measure of their involvement is grandparental childcare assistance (e.g., Ates 2017; Grundy et al. 2012; Xu et al. 2012). Grandparental involvement measures also include the frequency of contact between grandparent and grandchild (e.g., Bates and Taylor 2012, 2016; Danielsbacka and Tanskanen 2016; García-Campos et al. 2010), financial help, and other informal forms of assistance or emotional support (e.g., Fujiwara and Lee 2008). Also, grandparental health has been measured with various variables, such as self-rated health (e.g., Choi and Zhang 2018; Danielsbacka et al. 2019), longevity and time to death (Hilbrand et al. 2017a; Hilbrand et al. 2017b), cognitive functioning (e.g., Ahn and Choi 2019; Arpino and Bordone 2014), depression and mental health (e.g., Lee et al. 2019; Xu 2019), and functional limitations/abilities (e.g., O’Loughlin et al. 2017; Ku et al. 2012). Grandparental well-being has been measured with variables such as subjective well-being (SWB), perceived quality of life, happiness, and life satisfaction (e.g., Arpino et al. 2018; Conde-Sala et al. 2017; Danielsbacka and Tanskanen 2016; Nimrod 2008). In many cases, several measurements from both groups (health and well-being) are utilized in the same study.

Among non-coresiding grandparents, most of the results were positive (69%; 51/74). Meanwhile, only 19% (14/74) reported a negative association between grandparental involvement and well-being, while 14% (9/74) showed a negligible association (Table 2 and Appendix Table 8). The positive results were found from the data of European countries (e.g., Arpino and Bordone 2014; Mahne and Huxhold 2015), Asian countries (e.g., Luo et al. 2019; Park 2018), the USA (e.g., Hughes et al. 2007; Xu et al. 2017), and other countries (e.g., Grundy et al. 2012; Thiele and Whelan 2008). Thus, the positive results were not solely restricted to certain geographic regions. Furthermore, they were found among studies that contained cross-sectional data (e.g., Conde-Sala et al. 2017), longitudinal data (e.g., Di Gessa et al. 2016a), and methods for detecting causal relations [e.g., the IV approach (Arpino and Bordone 2014) or panel fixed-effect models (Danielsbacka et al. 2019)]. Since most studies utilize grandparental childcare support as an independent variable, this is the most common explanatory variable among the studies with positive results. Grandparental health or well-being were measured with several outcome variables, and thus, the positive associations were not restricted to certain health or well-being outcomes.

The negative results were most commonly accompanied with positive results (*n* = 9), and in these cases, the negative associations only applied to a certain grandparent group or outcome. Results that were solely negative were only found in five studies, which included associations between grandfathers’ frequency of contact with a grandchild and decreased life satisfaction (Sener et al. 2008), a grandparent’s centrality role and decreased psychological well-being (Muller and Litwin 2011), and grandparental childcare and increased depressive symptoms (Brunello and Rocco 2019).

Only nonsignificant results were found most likely among the studies that were not specifically focused on associations between grandparental involvement and well-being but considered a wider range of social connections or caregiving roles (i.e., caring for grandchildren was one measurement among others) (Hsu and Chang 2015; Nimrod 2008; O’Loughlin et al. 2017; Ward et al. 2019; Young and Denson 2014).

Among non-coresiding grandparent studies data used was representative in 65% of the studies (42/65) and non-representative in 35% of the studies (23/65). Among positive results 65% (33/51), among negative results 57% (8/14), and among nonsignificant results 67% (6/9), the data used were representative.

## Discussion and implications

The present review includes articles that consider the associations between grandparenting and grandparents’ health or well-being. In 68% of cases, custodial grandparenting was associated with decreased health or well-being of grandparents. Studies considering grandparents’ health or well-being who live in three-generation households provided mixed results (39% positive; 39% negative). The involvement of non-coresiding grandparents was associated with improved grandparental outcomes in 69% of the results. Thus, the most negative results were present in the case of custodial grandparents, the most mixed results were among those that involved grandparents living in three-generation households, and most positive results concerned the case of non-coresiding grandparents (Table 2).

We also considered whether the results were based on representative rather than non-representative data. Non-representative data were most commonly used in studies focusing on three-generation households (56%) whereas representative data were utilized mostly in studies of non-coresiding grandparents (65%). Among custodial grandparent studies, positive results were most commonly achieved with representative data (50%, 6/12), in three-generation households representative data constituted 60% (3/5) of nonsignificant results and in non-coresiding grandparent group also nonsignificant results were most likely conducted with representative data (67%, 6/9). Two last mentioned proportions are from the category that had overall lowest number of results.

However, when we consider *all* the results that were included in the studies in this review, the overall proportion of positive, negative and nonsignificant results appear different (Table 4). As in many studies, several results were investigated that were either due to a differentiation in the grandparent subgroups (e.g., the grandparents according to gender) or multiple outcomes, so the same study may include several positive, negative, or negligible results. When all the results were considered (*n* = 452), the most common in every grandparent group was nonsignificant. In the case of custodial grandparents, 44% (67/151) of the results were nonsignificant, 62% (53/85) in the case of three-generation households, and 51% (111/216) for non-coresiding grandparents (Table 4). Although the number and proportion of nonsignificant results increased after all the results were counted, the proportion of negative results remains the highest among custodial grandparents (43%), while that of the positive results were those of the non-coresiding grandparents (36%). However, it is evident that after counting all the results that address the association between grandparental involvement and grandparental health or well-being, the overall evidence for significant results (either positive or negative) becomes weaker.

**Table 4:** Summary of all associations in custodial, three-generation, and non-coresiding grandparent groups

	Positive association	Negative association	No association	Total no. of studies	Total no. of results
Custodial care	19 (13%)	65 (43%)	67 (44%)	34	151
Three-generation	15 (18%)	17 (20%)	53 (62%)	18	85
Non-coresiding	77 (36%)	28 (13%)	111 (51%)	65	216
Total	111 (25%)	110 (24%)	231 (51%)	117	452

Number of results and % of total number of results

Furthermore, as previously discussed, the associations that were found may not be causal in nature, but rather, they may reflect the selection of different caregiving groups. To observe how well the abovementioned studies capture the causal nature of the associations, we have compiled a table of the results that are only based on longitudinal data and/or methods that can detect causality. The ones that are most commonly utilized include panel fixed-effect models and instrumental variable approaches (Table 3). Overall, approximately 50% (59/117) of studies utilized longitudinal data and/or causal methods, and based on these, the most negative results were still found among custodial grandparents (57%; 12/21) and the positive among non-coresiding grandparents (72%; 26/36). However, a more detailed investigation reveals that only approximately 20% (22/117) of studies’ methods can actually address the question of causality (Table 5). Still, based on these studies, the negative effects were most commonly found among custodial grandparents (50%; 3/6) and three-generation households (60%; 3/5), while positive effects were found among non-coresiding grandparents (50%; 6/12).

**Table 5:** Summary of associations in custodial, three-generation, and non-coresiding grandparent groups with causal modeling

	Positive association	Negative association	No association	Total no. of studies	Total no. of results
Custodial care	2 (33%)	3 (50%)	1 (17%)	5	6
Three-generation	1 (20%)	3 (60%)	1 (20%)	5	5
Non-coresiding	6 (50%)	2 (17%)	4 (33%)	12	12
Total	9 (39%)	8 (35%)	6 (26%)	22	23

Number of results and % of total number of results

As was assumed, based on the hypothesis of Coall and Hertwig (2010, 2011), moderate grandparental involvement (e.g., that of non-coresiding grandparents) seems the most beneficial for grandparents, while negative effects were more common when grandparental involvement reached the highest level, like when grandparents became the primary caregivers for their grandchildren. However, as more detailed investigation has revealed, the most common result in all grandparenting contexts was nonsignificant. Also, although approximately half of the studies utilized longitudinal data, only one-fifth of the methods that were used could detect causal relations. This reveals two important questions: First, since there was a large number of nonsignificant results, is the interpretation concerning the association of grandparenting with grandparental health and well-being robust? Second, is the association causal? The first question could indicate a publishing bias, meaning that nonsignificant results may remain unpublished unless they are accompanied by at least one significant result. However, after all our robustness checks (accounting all results, distributing results based on causal methods and representativeness of data) the main finding remains the same: the most negative results are found among custodial grandparents and most positive results among non-coresiding grandparents.

Negative results found among custodial grandparents and grandparents living in three-generation households may reflect selection effects (concerning poorer health), as previously discussed. However, we also found negative results in the longitudinal data that was conducted with methods able to detect causal associations. At least in some circumstances, therefore, the interpretation that becoming a custodial grandparent or living in a three-generation household is detrimental for grandparental health or well-being seems to be robust.

In the case of three-generation households, it is important to consider the reason behind these living arrangements, as grandparental co-residence could either be a result of their poor health (i.e., they need daily support) or stem from a need to take care of their grandchildren. For the former, grandparents may receive significant support from their adult children, which can improve their health and well-being, but they are in poor health to begin with. Regarding the latter, the grandparents are likely in reasonably satisfactory health to begin with but may become constant “nannies” for their grandchildren, causing extra strain that may have a negative health effect.

One of our aims was to investigate whether the results in three groups of grandparents would differ according to study population, i.e., whether they were dependent on cultural context. Our prediction was that being a custodial grandparent or living in three-generation household could be less detrimental or even positive for grandparents in Asian countries. However, the positive/negative results regarding custodial grandparents or three-generation households were not solely related to the country of residence, although the results that were solely positive were more often found in studies with data from Asian countries (Chung and Park 2018; Guo et al. 2008; Ku et al. 2013; Tsai et al. 2013). Regarding non-coresiding grandparents, the positive results in this group were also found from the data of European countries, Asian countries, the USA, and other countries meaning that the positive results were not solely restricted to certain geographic regions. To conclude, we did find some support for the prediction that living with grandchildren would have less detrimental effects for grandparents in Asian countries, but also that the distribution of positive and negative results did not follow strictly the geographic or cultural distinctions.

Although an extensive and increasing number of studies have investigated whether grandparenting is associated with the health or well-being of grandparents, some gaps still exist in the research. While studies with longitudinal data are well-represented, more studies are needed that analyze the causal nature of the associations. Furthermore, studies using longitudinal data (especially with several follow-ups), and even those with causal methods, cannot disregard that a health decline is inevitable among older adults. Thus, studies should concentrate on the relative health decline rather than health improvement (e.g., Chen and Liu 2012). Thus, a hypothesis may be that moderately involved grandparents would suffer from a *slower* health decline than their counterparts. However, one problem with longitudinal designs is that they may suffer from selective attrition over time meaning that people who experience health decline drop out from the survey. Another relevant direction would include studies that use cross-sectional data and causal methods (e.g., instrumental variable approach), as they may capture the causal effect more accurately without involving the aging effect or selective attrition over time.

In several studies, grandparental outcomes have been separated by gender or ethnicity, so some additional segregation or interactions could be relevant. For instance, it is well-known that socioeconomic status is associated with health (e.g., Kim and Durden 2007), but few studies examine the interaction between socioeconomic status and grandparental involvement and its association with grandparental health or well-being (e.g., Chung and Park 2018; Mahne and Huxhold 2015). It is also well-known that lineage (i.e., whether a grandparent is from the maternal or paternal side) is strongly associated with grandparental childcare and being a custodial or coresiding grandparent (Tanskanen and Danielsbacka 2019). Still, surprisingly few studies consider this while studying the association between involved grandparenting and grandparental health or well-being (e.g., Danielsbacka and Tanskanen 2016). Number of grandchildren varies a lot across studies and also depends on the context of grandparenting. Custodial grandparents and grandparents living in three-generation household are commonly involved with grandchildren of one of their child whereas non-coresiding grandparents can be involved with grandchildren via several adult children. Not only the intensity of grandchild care but also the number of grandchildren to be cared for may affect grandparental outcomes. Thus, the number of grandchildren, especially the number of grandchildren via different children, is relevant factor to be considered in future studies.

Moreover, in the case of non-coresiding grandparents, the most common measure for grandparental involvement is childcare that is provided by grandparents. Looking after grandchildren without a parental presence may not capture all the positive aspects of being an involved grandparent. Thus, contact frequency or emotional closeness with grandchildren could be a more relevant measurement to understand the association between involved grandparenting and grandparental health or well-being. In addition, we have concentrated on studies that use the intensity of grandparental involvement as an explanatory variable but there are also other ways to compare grandparent types in respect to their health or well-being. For instance, the different styles of grandparenting (e.g., Neugarten and Weinstein 1964; Cherlin and Furstenberg 1985), different levels of reserves and strengths, or different role identities (e.g., Drew and Silverstein 2004) could lead different outcomes measured as grandparent health or well-being indicators.

The impact of grandparental involvement on grandparental health or well-being has been measured with varying measurements across the studies included in this review. This could be regarded as a limitation because the effects might differ regarding different outcomes. However, we did not find any clear biases on whether the associations would have been positive, negative, or negligible according to health or well-being measure used. This indicates that grandparental involvement may be similarly associated with various measures of health and well-being.

The question of how to increase the healthy years of one’s life is crucial in contemporary aging societies, so whether time spent with grandchildren could promote health or well-being remains relevant. Policy implications concerning this review’s findings are threefold. First, grandparents in custodial circumstances and three-generational households are the most vulnerable grandparent group, which policymakers should recognize. For them, caring responsibilities are not beneficial (although they are not solely detrimental either). Moreover, based on scant causal evidence, negative associations are not merely due to selection, which means that becoming or continuing a custodial/coresiding role as a grandparent could deteriorate health and well-being. However, especially in the case of these grandparent groups, cultural differences do exist and thus it is important to take into account the study population when the results are considered.

Moreover, among non-coresiding grandparents, their involvement is associated with improved health and well-being, although this association is not unequivocal. Despite the paucity of strong causal evidence, moderate grandparental involvement of non-coresiding grandparents should still be encouraged and enabled in terms of social policy decisions. Finally, we need more studies that can detect the causal nature of this association, as the lack of causal evidence concerns all three contexts of grandparenting.

## Appendix

See Tables 6, 7 and 8.

**Table 6:** Custodial care: studies concerning the association between custodial care arrangement and grandparents’ health and well-being (*n* = 34)

References	Population	Sample characteristics	Measure of grandparent's health/well-being	Association
Baker and Silverstein (2008a)	USA	8468; 52–74 years old; representative	Depressive symptoms	neg
Baker and Silverstein (2008b)	USA	5298 grandmothers; 50–70 years old; representative	Preventive health behavior	neg. (gms who recently began raising gc, less likely preventive health behav) & pos. (gms who had been raising and continue to raise gc, more likely preventive health behav)
Bigbee et al. (2011)	USA	485 grandmothers (rural–urban); non-representative	Physical and mental health	neg. (for rural gms in case of mental health)
Blustein et al. (2004)	USA	10,293 grandparents; 53–63 years old; representative	Depressive symptoms	neg
Bowers and Myers (1999)	USA	101 grandmothers (23 custodial, 33 part-time carers, 45 regularly visiting grandchildren); non-representative	Burden, parenting stress, grandparenting satisfaction, life satisfaction	neg
Chen et al. (2015)	USA	69,668; 50 + years old; representative	Frailty index (FI)	neg
Chen and Liu (2012)	China	14,954 person-year records; 55 and above; non-representative	Self-rated health (SRH)	ns
Choi and Zhang (2018)	South Korea	3092 grandmothers; 45 and above; representative	Self-rated health	ns
Chung and Park (2018)	South Korea	1948 grandmothers; 50–74 years old (in 2006); representative	Developmental trajectories of depressive symptoms and self-rated health over time	pos. (more depressive symptoms if stopped raising gc in low-income group)
Dunne and Kettler (2008)	Australia	52 caregiving and 45 non-caregiving grandparents (age-matched sample); non-representative	Stress, anxiety and depression scores	neg
Fuller-Thomson and Minkler (2000)	USA	79 African American grandparents who were raising a grandchild and 485 African American grandparents who had never been primary caregivers for a grandchild; non-representative	Depression (CES-D), activities of daily living	neg
Fuller-Thomson and Minkler (2005)	USA	319 American Indian or Alaska Native grandparent caregivers and 5956 AI/AN who reported that they were not caregivers to grandchildren; 45 years old and older; representative	Limitations in activities of daily living, functional disability, severe vision or hearing problem, poverty line status	neg
Goodman and Silverstein (2002)	USA	1058 grandmothers; non-representative	Grandmothers' well-being (negative affect, positive affect, life satisfaction, depression, and mental health)	neg. (Latino), pos. (African American), ns. (White)
Goodman and Silverstein (2006)	USA	1051 grandmothers; non-representative	Grandmothers' well-being (negative affect, positive affect, life satisfaction, and depression)	neg. (Latino), pos. (African American), ns. (White)
Hayslip et al. (1998)	USA	193 grandparents; non-representative	Psychosocial satisfaction and positive grandparental meaning	neg. (psychosocial satisfaction), pos. (positive grandparental meaning, men)
Hughes et al. (2007)	USA	12,872 grandparents; 50–80 years old; representative	Health behaviors (smoking, problem drinking, exercise, obesity) and mental and physical health (depressive symp., SRH, chronic conditions, functional limitations)	neg. (women: SRH (start), smoking (contin.)), pos. (women SRH (cont.))
Ice et al. (2010)	Kenya	287 Luo grandparents; 60 + years old; non-representative	BMI, glucose, hemoglobin, perceived health, mental health, systolic blood pressure (SBP)	neg. (perceived health, mental health; at *p* < 0.1: BMI)
Ice et al. (2012)	Kenya	640 Luo elders; 60 + years old; non-representative	Perceived and physiological measures of stress (cortisol levels and BP)	neg. (women: perceived stress), pos. (men: perceived stress)
Ice et al. (2008)	Kenya	287 Luo grandparents; 60 + years old; non-representative	Mental and physical health	pos. (women: vitality, nutritional status; men: mental health), neg. (men: nutritional status)
Komonpaisarn and Loichinger (2019)	Thailand	29,227 older people; 60–80 years old; representative	SRH, functional limitations, psychological well-being, happiness	pos. (custodial gp: functional limitations, SRH, psychological well-being), neg. (happiness)
Ku et al. (2013)	Taiwan	3711 grandparents; 50 + years old; representative	SRH; depressive symptoms; mobility limitations; life satisfaction	pos. (mobility limitations; recent custodial caregivers only, not long-term)
Minkler et al. (1997)	USA	3111 grandparents; representative	Depression levels	neg
Minkler and Fuller-Thomson (1999)	USA	173 custodial and 3304 non-custodial grandparents; representative	Summary measure of ADL limitations	neg
Minkler and Fuller-Thomson (2005)	USA	2362 African American grandparent caregivers with 40,148 non-caregiving peers; 45 + years old; representative	Functional limitations, limitations in ADL, income, and poverty	neg. (women: functional limitations, income, poverty)
Musil et al. (2011)	USA	485 grandmothers; non-representative	Caregiving stress and reward, intrafamily strain, social support, resourcefulness, depressive symptoms, mental and physical health, SRH, and perceived family functioning	neg. (stress, intrafamily strain, perceived family functioning, physical health, SRH, depressive symptoms, reward)
Musil (1998)	USA	90 grandmothers (58 had primary responsibility and 32 did not); 39–82 years old; non-representative	Health, depressed mood, anxiety, stress, coping, and social supports	neg. (stress [subscales: parent/child dysfunctional interaction, parenting distress], subjective and instrumental support)
Musil (2000)	USA	74 grandmothers living in the same home as grandchild(ren), 49 primary caregiver grandmothers, and 25 with partial/supplemental responsibility; 39–72 years old; non-representative	Self-assessed health, depression, parenting stress, anxiety, coping and social support	neg. (parenting stress [all subscales], instrumental support, depression) (no main effects on depression by caregiver status, but primary caregivers had higher time 2 depression scores)
Musil and Ahmad (2002)	USA	86 primary caregiver grandmothers, 85 partial/supplemental caregiver grandmothers in multigenerational homes, and 112 non-caregiver grandmothers; non-representative	Perceived stress, social support, self-assessed health, health problems, health visits, health maintenance, depressed mood	neg. (stress, instrumental support, self-assessed health, health problems, health visits), pos. (at * p* < 0.1: subjective support, depression)
Oburu and Palmerus (2005)	Kenya	241 caregiving grandmothers; non-representative	Stress levels	neg
Solomon and Marx (1999)	USA	11,591 women; 40 years and above; representative	Health status	neg
Strawbridge et al. (1997)	USA	42 grandparent, 44 spouse, and 130 adult–child caregivers with 1669 non-caregivers; 46–75 years old; representative	Mental and physical health (surveyed in 1974 and 1994) (symptoms of depression, happiness, self-reported health, and prevalence of chronic conditions or activity limitations)	neg. (happiness, chronic conditions, activity limitations; at * p* < 0.1: depression, SRH)
Szinovacz et al. (1999)	USA	1789 black and white grandparents; representative	Grandparents' subjective well-being (depressive symptoms and life satisfaction)	neg. (women: depressive symptoms) & pos. (men: depressive symptoms; gc leaving the household increase)
Wilmoth et al. (2018)	USA	2503 grandparents; non-representative	Well-being	neg
Yalcin et al. (2018)	Turkey	2563 women; 65 + years old; non-representative	Quality of life (SF-12; mental and physical), health status (Visual Analog Scale of EQ-5D, VAS) and symptoms of depression (Beck Depression Inventory, BDI)	neg

**Table 7:** Three-generation families: studies concerning the association between living in three-generation households and grandparents’ health and well-being (*n* = 18)

References	Population	Sample characteristics	Measure of grandparent's health/well-being	Association
Bigbee et al. (2011)	USA	485 grandmothers (rural–urban); non-representative	Physical and mental health	ns
Blustein et al. (2004)	USA	10,293 grandparents; 53–63 years old, representative	Depressive symptoms	neg
Chen et al. (2015)	USA	69,668 observations; 50 + years old; representative	Frailty index (FI)	neg
Chen and Liu (2012)	China	14,954 person-year records; 55 and above; non-representative	Self-rated health (SRH)	neg
Choi and Zhang (2018)	South Korea	3092 grandmothers; 45 and above; representative	Self-rated health	ns
Goodman (2003)	USA	987 grandmothers (512 custodial grandmothers and 475 coparenting grandmothers); non-representative	Depression and life satisfaction	ns
Goodman and Silverstein (2002)	USA	1058 grandmothers; non-representative	Grandmothers' well-being (negative affect, positive affect, life satisfaction, depression, and mental health)	neg. (African American), pos. (Latino), neg. (White)
Goodman and Silverstein (2006)	USA	1051 grandmothers; non-representative	Grandmothers' well-being (negative affect, positive affect, life satisfaction, and depression)	pos. (Latino), neg. (African American)
Guo et al. (2008)	China	1002 individuals; 50 years old and above; non-representative	Physical health status and mental health status	pos
Hsu and Chang (2015)	Taiwan	14,193 observations from 4731 older persons; 60 years old or above; representative	Happiness	ns
Hughes et al. (2007)	USA	12,872 grandparents; 50–80 years old; representative	Health behaviors (smoking, problem drinking, exercise, obesity) and mental and physical health (depressive symp., SRH, chronic conditions, functional limitations)	neg. (women: obesity (start + cont.); men: functional limitations (cont.), pos. (men: exercise)
Ku et al. (2012)	Taiwan	4582 adults; 50–80 years old at first interview; representative	SRH; depressive symptoms; mobility limitations	ns
Ku et al. (2013)	Taiwan	3711 grandparents; 50 + years old; representative	SRH; depressive symptoms; mobility limitations; life satisfaction	pos. (srh [both long-term and recent caregivers], mobility limitations [recent caregivers only], depressive symptoms [long-term only])
Musil et al. (2011)	USA	485 grandmothers; non-representative	Caregiving stress and reward, intrafamily strain, social support, resourcefulness, depressive symptoms, mental and physical health, SRH, and perceived family functioning	neg. (transitions to higher caregiving; physical health, stress, intrafamily strain, perceived family functioning), pos. (subjective support, instrumental support)
Musil (2000)	USA	74 grandmothers living in the same home as grandchild(ren), 49 primary caregiver grandmothers, and 25 with partial/supplemental responsibility; 39–72 years old; non-representative	Self-assessed health, depression, parenting stress, anxiety, coping and social support	neg. (parenting stress)
Musil and Ahmad (2002)	USA	86 primary caregiver grandmothers, 85 partial/supplemental caregiver grandmothers in multigenerational homes, and 112 non-caregiver grandmothers; non-representative	Perceived stress, social support, self-assessed health, health problems, health visits, health maintenance, depressed mood	neg. (stress, subjective support; at p ≤ 0.1: depression), pos. (instrumental support)
Tsai et al. (2013)	Taiwan	914 elders in 1993, 1792 elders in 1999 and 2292 elders in 2007; 60 years old and above; representative	Depressive symptoms	pos. (less symptoms if living with children (&gc))
Yalcin et al. (2018)	Turkey	2563 women; 65 + years old; non-representative	Quality of life (SF-12; mental and physical), health status (Visual Analog Scale of EQ-5D, VAS) and symptoms of depression (Beck Depression Inventory, BDI)	pos

**Table 8:** Non-coresiding care: studies concerning the association between grandparental involvement and grandparents’ health and well-being (*n *= 65)

References	Population	Sample characteristics	Type of grandparental	Measure of grandparent's	
			Involvement	Health/well-being	Association
Ahn and Choi (2019)	South Korea	27,947 observations for 8469 individuals; 45–84 years old; representative	Grandparents’ caregiving status	Cognitive functioning	pos
Arpino and Bordone (2014)	12 countries (Austria, Belgium, Denmark, France, Germany, Greece, Israel, Italy, Spain, Sweden, Switzerland, and the Netherlands)	5610 women and 4760 men; 50–80 years old; representative	Childcare (how often on average they cared for grandchild during last 12 months (5 point scale), hours per day grandparents look after grandchildren)	Verbal fluency, numeracy, delayed recall, immediate recall, orientation	pos. (verbal fluency)
Arpino et al. (2018)	20 countries (Austria, Belgium, Croatia, Czech Republic, Denmark, Estonia, France, Germany, Greece, Hungary, Israel, Italy, Luxemburg, the Netherlands, Poland, Portugal, Slovenia, Spain, Sweden, and Switzerland)	83,427 observations of 42,868 individuals; 50–84 years old; representative	Childcare (and being a grandparent, number of grandchildren, how often they engaged in grandchild care)	Subjective well-being (SWB)	pos
Arpino and Gómez-León (2019)	12 countries (Austria, Germany, Sweden, Netherlands, Spain, Italy, France, Denmark, Greece, Switzerland, Belgium, Israel)	5012 men and 6784 women; 50–84 years old; representative	Combination of grandchild care with other care roles	Depressive symptoms	pos. (grandmothers, only gc care—no other care)
Ates (2017)	Germany	1875 observations of 625 individuals; 40 years and above; representative	Childcare	Self-rated health (SRH)	ns
Bates and Taylor (2012)	USA	351 grandfathers; non-representative	Grandfather involvement (contact frequency, generative activities, commitment)	Mental health	pos
Bates and Taylor (2016)	USA	351 grandfathers; non-representative	Grandfather involvement (contact frequency, generative activities, commitment)	Mental health	pos
Bigbee et al. (2011)	USA	485 grandmothers (rural–urban); non-representative	rural/urban effects, caregiver status	Physical and mental health	ns
Bowers and Myers (1999)	USA	101 grandmothers (23 custodial, 33 part-time carers, 45 regularly visiting grandchildren); non-representative	Level of caregiving (full-time, part-time, no care)	Burden, parenting stress, grandparenting satisfaction, life satisfaction	pos. (gp satisfaction [part-time vs. non-caregiving), neg. (burden, parenting stress [full-time vs part-time], life satisfaction [full-time vs non-caregiving])
Brunello and Rocco (2019)	12 European countries (Austria, Germany, Sweden, Netherlands, Spain, Italy, France, Denmark, Switzerland, Belgium, the Czech Republic, and Poland)	13,091 (7397 females and 5694 males); 50 years and above; representative	Childcare (hours per month)	Depressive symptoms	neg
Burn et al. (2014)	Australia	186 women; 57–68 years old; non-representative	Childcare	Cognition	neg. (high level of childcare), pos. (low-level of childcare)
Burn and Szoeke (2015)	Australia	224 women; 65 + years old, non-representative	Childcare	Cognitive function	pos
Chen et al. (2015)	USA	69,668 observations; 50 + years old; representative	Grandparents’ living arrangements and in case of non-coresiding grandparents: amount of childcare	Frailty index (FI)	pos
Choi et al. (2013)	14 countries (Denmark, Sweden, Austria, France, Germany, Switzerland, Belgium, the Netherlands, Spain, Italy, Greece, Israel, Czech Republic, and Poland)	7,238; 60 + years old; representative	Five types of productive activities (paid work, formal volunteering, caregiving, informal helping and caring for grandchildren)	Depression	ns
Choi and Zhang (2018)	South Korea	3092 grandmothers; 45 and above; representative	Grandparenting type and transition and grandparenting intensity	Self-rated health	pos. (overall nonresidential grandparenting)
Conde-Sala et al. (2017)	15 countries (Denmark, Sweden, The Netherlands, Switzerland, Luxembourg, Austria, Germany, Belgium, France, Slovenia, Czech Republic, Estonia, Spain, Italy, and Israel)	33,241; 65 + years old; representative	Sociodemographic, socioeconomic factors, physical exercise and activities (including grandparenting), physical health, depressive symptoms, life expectancy and healthy life expectancy, suicide rate, gross domestic product (GDP) per capita based on purchasing power parity (PPP)	Perceived quality of life	pos. (all countries combined & Mediterranean country cluster: caring for gc (yes/no) – > better perceived QoL)
Danielsbacka et al. (2019)	11 European countries (Austria, Germany, Sweden, Netherlands, Spain, Italy, France, Denmark, Switzerland, Belgium, and Czech Republic)	41,713 person-observations from 24,787 persons; 50 and above; representative	Grandchild care for < 14-year-old grandchildren	SRH; difficulties with activities of daily living (ADLs); depressive symptoms; life satisfaction; meaning of life	pos. (ADL)
Danielsbacka and Tanskanen (2016)	Finland	2152; 62–67 years old; representative	Contact frequencies with grandchildren	Grandparental happiness	pos. (maternal grandmothers, higher contact with gc – > happier)
Di Gessa et al. (2016a)	10 European countries (Austria, Belgium, Switzerland, Germany, Denmark, Spain, France, Italy, the Netherlands, and Sweden)	8485 people; 50 and above; representative	Intensive and non-intensive grandparental childcare	Subsequent health (self-rated health, depressive symptoms, and disability)	pos
Di Gessa et al. (2016b)	11 European countries (Austria, Belgium, Switzerland, Germany, Denmark, Spain, France, Italy, Greece, the Netherlands, and Sweden)	8972 grandmothers and 6567 grandfathers; 50 years and above; representative	Intensive and non-intensive grandparental childcare	Latent continuous physical health variable based on self- and observer-measured indicators	pos. (grandmothers, both intensive and non-intensive care)
Fujiwara and Lee (2008)	USA	724 adults; 25–74 years old; representative	Altruistic behaviors for children and grandchildren (ABC) (informal assistance, emotional support, financial support)	Major Depression (MD)	pos. (men: informal assistance 1–10 h/month, and financial support 1–50 $/month), neg. (women, at * p* < 0.1: financial support 1–50 $/month)
García-Campos et al. (2010)	Mexico	386 postmenopausal women; 55–75 years old; non-representative	Number of children and grandchildren and frequency of their contact (in addition: age, date of the last menstrual period, age at menarche, previous menstrual history, number of pregnancies and deliveries, height, weight and waist and hip circumferences, BMI, waist/hip ratio, schooling in years, work, hours of exercise per week, alcohol consumption, smoking habit)	Women’s symptoms at postmenopause—hot flushes, vaginal dryness, depressive mood, anxiety, non-specific symptoms of depression (NSSD; problems with digestion, loss of sexual interest, and weight loss), empty nest syndrome (ENS)	neg. (caring for gc: loss of sexual interest, depression, NSSD, and ENS; meeting gc: ENC)
Grundy et al. (2012)	Chile	2000 people; 66–68 years old; representative	Hours per week of grandchild care	Mental well-being two years later (life satisfaction, depression, Mental Component Summary (MCS) SF-36	pos. (gf life satisfaction), pos. (gm less depression)
Guo et al. (2008)	China	1002 individuals; 50 years old and above; non-representative	Whether the respondent currently helps with childcare; if the respondent lives with any grandchildren	Physical health status and mental health status	pos
Hilbrand et al. (2017a)	Germany	516 older adults; representative	grandparenting and supporting others in the social network	Longevity	pos
Hilbrand et al. (2017b)	Germany	516 older adults; representative	frequency of childcare	Time to death	pos
Hsu and Chang (2015)	Taiwan	14,193 observations from 4731 older persons; 60 years old or above; representative	Social connection variables included living arrangements, contacts with children/grandchildren/parents/relatives/friends, telephone contacts, providing instrumental and informational support, receiving instrumental and emotional support, and social participation	Happiness	ns
Hughes et al. (2007)	USA	12,872 grandparents; 50–80 years old; representative	Caring for grandchildren	Health behaviors and mental and physical health	pos. (grandmothers: exercise & continued care; SRH & started care + continued care), pos. (gf: exercise & started care)
Jun (2015)	South Korea	2341 female; 45–74 years old at time 2; representative	Grandchild care	Cognitive functioning	pos. (for higher educated, both instantaneous and lagged effect)
Kim et al. (2017)	South Korea	5129 grandparents; 50 years old and above without depression; representative	Intensity of grandchild care (hours spent caring for a grandchild per week)	Depressive symptoms	pos
Komonpaisarn and Loichinger (2019)	Thailand	29,227 older people; 60–80 years old; representative	Grandchild care for < 10-year-old grandchildren	SRH; functional limitations; psychological well-being; happiness	neg. (self-rated health, functional limitations, and psychological well-being)
Ku et al. (2012)	Taiwan	4582 adults; 50–80 years old at first interview; representative	Grandparental childcare	SRH; depressive symptoms; mobility limitations	pos.(better SRH & less depressive symptoms; only significant for grandparents receiving financial support from adult children)
Ku et al. (2013)	Taiwan	3711 grandparents; 50 + years old; representative	Grandparental caregiving status (non-caregivers; three-generational; custodial; non-coresiding caregivers)	SRH; depressive symptoms; mobility limitations; life satisfaction	pos. (better SRH & less mobility limitations for long-term non-coresiding caregivers), BUT ns. in FE-models (for nonresidential gps), ns. (depressive symptoms and life satisfaction; for recent caregivers, all outcomes)
Lee et al. (2019)	South Korea	922 grandparents; 65 years old or above; non-representative	Grandparental childcare	Depression scores, suicidal ideation	pos. (moderate care and less depression) pos. (moderate and high care and less suicidal ideation)
Luo et al. (2019)	China	13,596; 50 + years old; representative	Caring for grandchildren	Cognitive decline	pos
Mahne and Huxhold (2015)	Germany	990 grandparents; mean age 74 years; representative	Relationship quality with children and grandchildren (measured with 2 variables: contact frequency and emotional closeness)	Subjective well-being (SWB), measured with life satisfaction, positive affect, negative affect, loneliness	pos. (life satisfaction, positive affect, negative affect (reduces), loneliness (reduces))
Mansson (2014)	USA	104 grandparents; 60–91 years old; non-representative	Grandparents' expressions of affection (4 types: love and esteem, caring, memories and humor, and celebratory)	Psychological health (self-reported stress, loneliness, self-reported general mental health)	pos. (stress, mental health)
Markides and Krause (1985)	USA	1125 Mexican Americans; 65–80 years old; representative	Intergenerational solidarity (association and affection)	Psychological well-being—Life Satisfaction Index, Center for Epidemiologic Studies Depression (CES-D) scale	pos. (self-perceived affection with gc and gp life satisfaction)
McGarrigle et al. (2018)	Ireland	8504 people; 50 + years old; representative	Grandchild care (numbers of hours of grandchild care reported in the past month)	depressive symptoms and quality of life	neg. (those with primary education or lower and no active or social leisure: intensive care, both outcomes), pos. (tertiary-educated gp with no active or social leisure: intensive care, quality of life) (secondary-educated gp with active and social leisure: low-intensity care, quality of life)
Mellqvist et al. (2011)	Sweden	80 suicide attempters; 70 years old and above; non-representative	Social (e.g., too little time spend with grandchildren) and health variables	Sense of coherence (SOC)	pos. (too little time spend with children and grandchildren were both associated positively with lower SOC meaning that more time with them would most likely associate positively)
Monin et al. (2014)	USA	2025 U.S. veterans; 60 years old or above; representative	Age, gender, education, marital status, income, combat exposure, caregiving hours, caring for a grandchild or other, and scores on physical health, psychological health, cognitive functioning, the positive psychological factor, and social support factor	Physical strain, emotional strain, and reward	pos. (reward), neg. (physical strain) NOTE: comparing only gp caregivers to other types of caregivers
Moore and Rosenthal (2015)	Australian	1205 grandmothers; 34–92 years old; non-representative	personal resources (age, health, education, being partnered) and grandmother engagement (number of grandchildren, hours/week spent with them, frequency of activities with grandchildren, grandmother satisfaction)	Generativity, life satisfaction, grandmother satisfaction	pos. (frequency of activities: generativity, gm satisfaction) (only correlation, ns. in regression: frequency of activities and life satisfaction; hours/week and gm satisfaction), ns. (hours/week: generativity and life satisfaction)
Muller and Litwin (2011)	11 European countries (Denmark, Sweden, Austria, Germany, France, Switzerland, Netherlands, Belgium, Spain, Italy, and Greece)	3888 grandparents; 50 years old and above; representative	Grandparent role centrality (calculated with standardized frequency of contact, the summary score for beliefs on grandparenting, and the grandparent-focused role occupancy measure and summed)	Psychological well-being (depressive symptoms)	neg. (the more gp role centrality the more depressive symptoms)
Neuberger and Haberkern (2014)	14 European Countries (Austria, Belgium, Czech Republic, Denmark, France, Germany, Greece, Ireland, Italy, the Netherlands, Poland, Spain, Sweden, and Switzerland)	12,740 grandparents; 50 years and above; representative	Grandchild care, grandparent obligations	Quality of life	pos. (high gp oblig. & gp care – > better QoL)
Nimrod (2008)	Israel	383 recently retired individuals; 50 years old and above; non-representative	Get-togethers (grandchildren as one group)	Life satisfaction	ns. (concerning gc)
O'Loughlin et al. (2017)	Australia	1261 men and women; 60–64 years old; representative	Caregiving status (giving care to grandchildren or other family member/friend, yes/no and hours per week)	Mobility difficulties, self-rated health, subjective well-being (life satisfaction, quality of life)	ns. (grandchild care, all outcomes)
Park (2018)	South Korea	255 grandparents; non-representative	Grandparenting role type, involvement level, mediating effect of care burden (stress)	Psychological well-being	pos
Sener et al. (2008)	Turkey	200 persons; 60 + years old; non-representative	Socioeconomic, demographic (age, education, marital status, income, perceived healthiness, and physical distance from adult children), and relational frequency of contact with loved ones (children, grandchildren, siblings, and friends) and relational satisfaction	Life satisfaction	neg. (men; frequency of contact with gc)
Sheppard and Monden (2019)	15 countries (Austria, Belgium, Czechia, Denmark, Estonia, France, Germany, Italy, the Netherlands, Portugal, Slovenia, Spain, Sweden, Switzerland, and Israel)	13,506 respondents (3511 (26%) transitioned to grandparent status); 50 years old and above; representative	Caring for grandchildren, (becoming a grandparent)	Depression, life satisfaction, subjective life expectancy	ns. (gc care and outcomes)
Sobol and Ben-Shlomo (2019)	Israel	197 first time grandparents; non-representative	Age, SES, education, gender, economic stress, work commitment, grandchild care burden, self-mastery, family support	Grandparents' mental health, grandparents' personal growth	pos. (gc care burden & personal growth)
Szinovacz and Davey (2006)	USA	1200 grandfathers and 1481 grandmothers; 51–60 years old at wave 1, representative	Retirement and grandchild care obligations	Well-being (depressive symptoms)	neg. & pos. (gms; retired gms & extensive care – > neg; working gms & extensive care – > pos)
Tang et al. (2016)	USA	2365 older adults (818 designated caregivers); 60 years old and above; non-representative (representative only of Chinese-American older adults in a large metropolitan area)	Grandparent caregiver status, caregiving time, burden, pressure, and perceived negative effect in caregivers	Psychological well-being (depressive symptoms, anxiety, stress, and loneliness)	pos. (being a caregiver: all outcomes)
Thiele and Whelan (2008)	Australia	149 non-custodial grandparents; up to 80 years old; non-representative	Weekly childcare contact with grandchildren, grandparental meaning, generativity	Grandparent satisfaction	pos. (childcare hours – > valued elder meaning, gp satisfaction [correlation only])
Thomas (1986)	USA	177 grandmothers and 105 grandfathers; 45–90; non-representative	Characteristics of grandparents' families (grandchildren's number and proximity, and ages of oldest and youngest grandchildren), grandparent characteristics (age, gender, marital status, and retirement status), and perceived responsibility scores (disciplining, caretaking, helping, and advising)	Grandparenting satisfaction	pos. (cg care)
Triadó et al. (2014)	Spain	312 grandparents; 46–91; non-representative	Socio-demographic variables (grandparent and grandchildren genders, ages, and family lines), indicators of intensity of care, the types of care provided, evaluation of behavioral problems in the grandchildren, satisfaction, and difficulties with care responsibilities	Number of health problems, perceived health, and satisfaction with life	neg. (time since care began – > perceived health), pos. (hours per week – > life satisfaction [ns. after controlling for difficulties with care])
Tsai (2016)	Taiwan	2930 grandparents; 50 years old and above, representative	Elders’ changing behavior in caring for grandchildren from 2003 to 2007, age, gender, educational level, work status and self-reported health status in 2007	Changes in depression symptoms from 2003 to 2007	pos. (less depressive symptoms)
Tsai et al. (2013)	Taiwan	914 elders in 1993, 1792 elders in 1999 and 2292 elders in 2007; 60 years old and above; representative	Providing grandchild care	Depressive symptoms	pos. (providing no gc care – > greater risk for depression or feeling lonely)
Ward et al. (2019)	Ireland	3646 respondents; representative	Gender, education and whether respondents lived with a partner, self-rated health, social connectedness, household income	Quality of life (QoL)	ns. (caring for gc was associated with QoL in baseline but not in longitudinal analysis)
Wood and Robertson (1978)	USA	257 grandparents; mean age 65; non-representative	Grandchildren, friendship and organizations involvement	Morale (life satisfaction)	pos. (association between gc involvement and life satisfaction)
Xu et al. (2017)	USA	2775 grandparents; 60 years old and above; representative	Grandparent caregiving time (hours/week), caregiving burden, and caregiving pressure	Psychological well-being (depressive symptoms and quality of life)	pos. (depressive symptoms)
Xu et al. (2012)	China	1704 caregivers; 60 years old and above, representative	Grandparent caregiving intensity	life satisfaction	pos
Xu (2019)	China	2663 to 3770; representative	Grandparents’ self-reported family caregiving in the past year, gender and rural–urban residence	Mental health (life satisfaction and depressive symptoms) and physical health (levels of high sensitivity C-reactive protein (CRP; chronic inflammation and acute infection), hypertension, high-risk pulse rate, and diabetes)	pos. (urban grandfathers: depressive symptoms [at * p* < 0.1]; urban grandmothers: life satisfaction and chronic inflammation), neg. (rural grandfathers: high-risk pulse)
Yalcin et al. (2018)	Turkey	2563 women; 65 + years old, non-representative	Study vs. control group, age, education, income, number of children and grandchildren, mean number of grandchildren cared for, mean age of grandchildren receiving care, mean time spent on grandchild car, mean length of care per week, and length of day- and nighttime care	Quality of life (SF-12; mental and physical), health status (Visual Analog Scale of EQ-5D, VAS) and symptoms of depression (Beck Depression Inventory, BDI)	pos. (mean time spent caring for grandchildren per week – > BDI; mean duration of daytime care & mean duration of nighttime care – > SF-12 [mental and physical component], VAS), neg. (time spent caring for grandchildren to date – > BDI, VAS; mean time spent caring for grandchildren per week – > SF-12 [both components], VAS; mean duration of daytime care – > BDI; mean duration of nighttime care – > BDI)
Young and Denson (2014)	Australia	148 non-custodial Australian boomer grandparents; non-representative	Amount of time spent engaged in, desire to change that time, and satisfaction with four different roles: grandchild care, paid work, other family care, and home duties	Psychosocial distress (symptoms of nervousness, agitation, psychological fatigue, and depression), self-esteem	ns. (time spent providing care: both outcomes)
Zhang et al. (2015)	China	3418 elderly respondents; representative	Social engagement (social activity, productive activity)	SRH, PWB	pos. (whole sample, men, and the “young-old”: both outcomes)
